# Mortality risk factors in community-dwelling, subjectively healthy, Swiss older adults: update after 8-years follow-up

**DOI:** 10.1186/s12877-023-03959-2

**Published:** 2023-05-17

**Authors:** Jean-Pierre Gutzwiller, Krisztina Müller-Bolla, Carlo Ferrari, Zeno Stanga, Urs E. Nydegger, Lorenz Risch, Martin Risch

**Affiliations:** 1 Magendarm Thalwil AG, Zürcherstrasse 61, Thalwil, 8800 Switzerland; 2 Department of Diabetes, Endocrinology, Nutritional Medicine and Metabolism, Bern University Hospital and University of Bern, Bern, Switzerland; 3 Università degli Studi di Milano, Milano, Italy; 4 Divisions of Clinical Chemistry and Haematology, Labormedizinisches Zentrum Dr. Risch, Waldeggstrasse 37, Liebefeld, Bern 3097 Switzerland; 5 University of Basel, Klingelbergstrasse 61, Basel, 4056 Switzerland; 6 Department of General Internal Medicine, Bern University Hospital and University of Bern, Bern, Switzerland; 7 Labormedizinisches Zentrum Dr. Risch, Landstrasse 157, Schaan, 9494 Liechtenstein; 8 Central Laboratory, Kantonsspital Graubünden, Loëstrasse 170, Chur, 7000 Switzerland

**Keywords:** Osteoporosis, Mortality, Diabetes, Hypertension, Pensioners, Switzerland

## Abstract

**Background:**

Worldwide population is ageing, but little is known regarding risk factors associated with increased mortality in subjectively healthy, community-dwelling older adults. We present the updated results of the longest follow-up carried out on Swiss pensioners and we provide results on potential risk factors associated with mortality before the onset of the COVID-19 pandemic.

**Materials and methods:**

Within the SENIORLAB study, we collected demographic data, anthropometric measures, medical history, and laboratory parameters of 1467 subjectively healthy, community-dwelling, Swiss adults aged ≥ 60 years over a median follow-up of 8.79 years. The variables considered in the multivariable Cox-proportional hazard model for mortality during follow-up were selected based on prior knowledge. Two separate models for males and females were calculated; moreover, we fitted the old model obtained in 2018 to the complete follow-up data to highlight differences and similarities.

**Results:**

The population sample included 680 males and 787 females. Age of participants ranged between 60 and 99 years. We experienced 208 deaths throughout the entire follow-up period; no patients were lost at follow-up. The Cox-proportional hazard regression model included female gender, age, albumin levels, smoking status, hypertension, osteoporosis and history of cancer within predictors of mortality over the follow-up period. Consistent findings were obtained also after gender stratification. After fitting the old model, female gender, hypertension, and osteoporosis still showed statistically significant independent associations with all-cause mortality.

**Conclusions:**

Understanding the predictors of a healthy survival can improve the overall quality of life of the ageing population and simultaneously reduce their global economic burden.

**Trial registration:**

The present study was registered in the International Standard Randomized Controlled Trial Number registry: https://www.isrctn.com/ISRCTN53778569 (registration date: 27/05/2015).

## Introduction

Worldwide population is ageing, and the number of older people is expected to double by 2050 [1]. Since 1971, the Swiss Federal Office of Statistics has reported a steadily rising trend in the median age of the indwelling population [2]. In 2020, more than 1.5 million Swiss people were aged over 65 years old, with an old-age dependency ratio of 30.7 [3]. This reflects into budgetary pressure on the entire society because it offsets the balance between income-producing subjects and resource-consuming elderly people [4].

Although elderly often perceive themselves as healthy, their health status is a complex network of pathophysiological conditions and controlled comorbidities constantly influencing each other. Even the slightest imbalance of this delicate equilibrium may cause the progression of comorbidities, eventually leading to death [5]. Understanding predictors of mortality can improve the overall quality of life of the ageing population and simultaneously reduce their global economic burden.

Based on these assumptions, since the late 90s, the interest of the scientific community has focused on unveiling potential risk factors associated with mortality in community-dwelling older adults. The cornerstone is represented by the Cardiovascular Health Study, published in 1998 by Fried and colleagues [5]. The strength of this project was to be the first to perform an analysis on several detailed clinical parameters and values that were not routinely measured in the outpatient setting. Among the 78 characteristics assessed in the study, 20 of them were significantly and independently associated with increased in 5-year mortality risk. These variables ranged from older age or male gender to smoking history and narrow ankle-brachial pressure index [5]. Among laboratory values, elevated fasting glucose, low albumin and elevated creatinine levels were characterized by a two- to five-fold increased risk of mortality [5]. Notably, neither osteoporosis nor history of cancer were associated with mortality in this cohort of subjects [5].

Twelve years later, the Rotterdam study reached similar conclusions regarding predictors of survival [6]. Moreover, the same authors concluded that almost all variables predictive of total survival were also predictive of morbidity-free survival, providing potential interventional targets to guarantee a healthy ageing of the population.

In 2018 we published a cohort study evaluating the impact of several clinical and serological parameters in subjectively healthy, community-dwelling, older Swiss adults over a 4-year follow-up period [7]. Our findings highlighted osteoporosis, diabetes and hypertension as major risk factors for mortality in more than 1400 Swiss adults aged 65 years or older. Osteoporosis-related bone fractures often lead to serious disability, chronic pain, and increased premature mortality [8, 9], while diabetes mellitus has been acknowledged as the ninth major cause of reduced life expectancy [10], mostly related to its microvascular complications, which may play a major role in the onset of cardiovascular morbidity [11]. Moreover, hypertension is responsible for 50% of cardiovascular or cerebrovascular events worldwide [12]. Therefore, our results were clinically meaningful and supported by evidence retrieved from the literature.

Four years later, we decided to update the results of this prospective cohort study by adding new mortality data from the extended follow-up period. In particular, we wanted to explore whether osteoporosis, diabetes and hypertension still played a role as predictors of all-cause mortality in our cohort over a longer time frame. Moreover, we aimed at identifying new predictors of all-cause mortality in the older Swiss population during a follow-up of more than 8 years.

## Materials and methods

### Study population and design

The SENIORLAB study is an observational, prospective cohort study conducted on 1467 subjectively healthy residents of Switzerland aged ≥ 60 years [13]. Details regarding aim, study design and settings, patients’ characteristics and data collection were already described in detail elsewhere [7, 13]. Briefly, all patients provided demographic data, anthropometric measures, and a fasting blood sample on which more than 110 laboratory parameters were measured. Medical history, comorbidities and current medications were mostly assessed by direct reporting during initial interview of each participant, or by means of official medical reports (whenever available). Positive history of cancer was considered in case of subjects diagnosed with a malignant disease in the 6 years preceding the inclusion in the study. Vital status was assessed by email or telephonic follow up every 3 to 5 years by directly contacting the participants of the study, their relatives, or caregivers. Assessment of death events and causes (whenever available) was conducted on behalf of the public authorities. Final data for analysis were retrieved from the SENIORLAB database on December 31st, 2019. Ethical approval for the present study was obtained from the Cantonal Ethics Committee of Bern (KEK Bern, Study Nr 166/08), Bern, Switzerland.

### Statistical analysis

To describe our population sample, continuous variables are presented as mean ± standard deviation (SD) or median and interquartile range (IQR) as appropriate; categorical variables are reported as absolute numbers and percentages. The selection of candidate variables to be included in the Cox proportional hazard regression analysis was performed based on prior clinical knowledge and after a thorough literature search. These included sex, age, overweight (BMI > 25 kg/m^2^), albumin (as a proxy of nutritional status), smoking, diabetes, hypertension, history of cancer, renal disease and osteoporosis. As there was a significant interaction between age and overweight (which is also known from the literature [14]), we introduced separate age variables for normal and overweight subjects.

The proportional hazard assumption was assessed using Schoenfeld’s test. As it was violated (p = 0.002), we considered all hazard ratios (HRs) as time-dependent, and alternatively used one of the three time-functions *t*, *natural logarithm of t* and *square root of t* to define interaction terms between the predictor variables and time.

We then conducted a backward selection of the time-interactions based on AIC. For variables with a remaining time-dependency of the HR, the HR reported refers to a follow-up time of 4 years. For these variables, HR(t) is represented graphically.

The model was also stratified by sex and gender-differences in HRs were assessed using the Chi-squared test. Kaplan-Meier plots were generated for diabetes and osteoporosis. For comparison, we also fitted our previous model obtained after 4 years of follow-up [7] to the extended follow-up data.

Statistical analysis was performed using STATA® ver. 16.0 (Stata Corp., 2000, College Station, TX, USA) and MedCalc® (MedCalc Software bv, 2020, Ostend, Belgium) for Windows.

## Results

### Population characteristics

Baseline clinical characteristics of the study participants are summarized and stratified by sex in Table 1. Continuous variables are presented as mean ± SD; categorical variables are reported as absolute numbers and percentages. Overall, the population sample included 1467 subjects, of which 680 (46.3%) males and 787 (53.7%) females. The mean age of participants was 72.1 years (± 7.8), ranging from 66 to 99 years old.

Gender comparison revealed higher BMI, serum creatinine, cystatin C level, and insulin resistance index (HOMA-IR) in men, while average 25-OH vitamin D levels were higher in women. Type 2 diabetes mellitus (T2DM), hypertension and active smoking were more common in the male population, while rheumatologic disorders, osteoporosis and depression syndromes were more prominent in females (Table 1).

By 31st December 2019, our population experienced 208 deaths (14.2%) throughout the entire follow-up period, of which 106 (7.2%) among males and 102 (7.0%) among females. No patients were lost to follow-up and the vital status could be retrieved for the entire cohort.

**Table 1:** Clinical characteristics of the participants (N = 1467)

	Overall population	Males (N = 680)	Females (N = 787)
Age (years, mean ± SD)	72.1 (± 7.8)	71.7 (± 7.6)	72.4 (± 8.0)
BMI (kg/m^2^, mean ± SD)	25.5 (± 3.8)	26.1 (± 3.5)	24.9 (± 4.0)
CRP (mg/l, mean ± SD)	2.7 (± 6.0)	2.8 (± 5.7)	2.6 (± 6.3)
Crea (mg/dl, mean ± SD)	77.0 (± 19.5)	87.0 (± 18.8)	68.3(± 15.6)
CysC (mg/l, mean ± SD)	0.8 (± 0.2)	0.9 (± 0.2)	0.8 (± 0.2)
Albumin (g/l, mean ± SD)	43.2 (± 2.6)	43.2 (± 2.7)	43.2 (± 2.5)
HOMA-IR (mean ± SD)	2.5 (± 6.5)	3.1 (± 9.5)	2.1 (± 1.6)
Hb1Ac (%, mean ± SD)	5.9 (± 0.6)	5.9 (± 0.7)	5.9 (± 0.5)
25-OH vitamin D (mmol/l)	23.9 (± 11.0)	23.2 (± 10.4)	24.6(± 11.3)
Diabetes, n (%)			
Yes	70 (4.8%)	52 (7.7%)	18 (2.2%)
No	1397 (95.2%)	628 (92.3%)	769 (97.8%)
Hypertension, n (%)			
Yes	562 (38.3%)	285 (41.9%)	277 (35.2%)
No	905 (61.7%)	395 (58.1%)	510 (64.8%)
Renal disease, n (%)			
Yes	36 (2.5%)	18 (2.6%)	18 (2.3%)
No	1431 (97.5%)	662 (97.4%)	769 (97.7%)
History of neoplasia, n (%)			
Yes	106 (7.2%)	48 (7.1%)	58 (7.4%)
No	1361 (92.8%)	632 (92.9%)	729 (92.6%)
Pulmonary disease, n (%)			
Yes	22 (1.5%)	10 (1.5%)	12 (1.5%)
No	1445 (98.5%)	670 (98.5%)	775 (98.5%)
Neurological disorders, n (%)			
Yes	263 (17.9%)	127 (18.7%)	136 (17.3%)
No	1204 (82.1%)	553 (81.3%)	651 (82.7%)
Smoking (active), n (%)			
Yes	100 (6.8%)	63 (9.3%)	37 (4.7%)
No	1367 (93.2%)	617 (90.7%)	750 (95.3%)
Rheumatologic disorders, n (%)			
Yes	154 (10.5%)	56 (8.2%)	98 (12.5%)
No	1313 (89.5%)	624 (91.8)	689 (87.5%)
Bleeding disorders, n (%)			
Yes	14 (0.9%)	8 (1.2%)	6 (0.8%)
No	1453 (99.1%)	672 (98.8%)	781 (99.2%)
Osteoporosis, n (%)			
Yes	53 (3.6%)	3 (0.4%)	50 (6.3%)
No	1414 (96.4%)	677 (99.6%)	737 (93.7%)
Depression syndrome, n (%)			
Yes	30 (2.0%)	4 (0.6%)	26 (3.3%)
No	1437 (98.0%)	676 (99.4%)	761 (96.7%)
Medications, n (%)			
≤ 2	1170 (79.7%)	512 (75.3%)	658 (83.6%)
3–5	293 (20.0%)	165 (24.3%)	128 (16.3%)
≥ 6	4 (0.3%)	3 (0.4%)	1 (0.1%)

BMI: body mass index, CRP: C reactive protein, Crea: serum creatinine, CysC: serum cystatin C, HOMA-IR: homeostatic model assessment for insulin resistance, Hb1Ac: glycated haemoglobin, COPD: chronic obstructive pulmonary disease, SD: standard deviation

### Multivariate analysis

#### Overall and sex-specific models

**Fig. 1:**
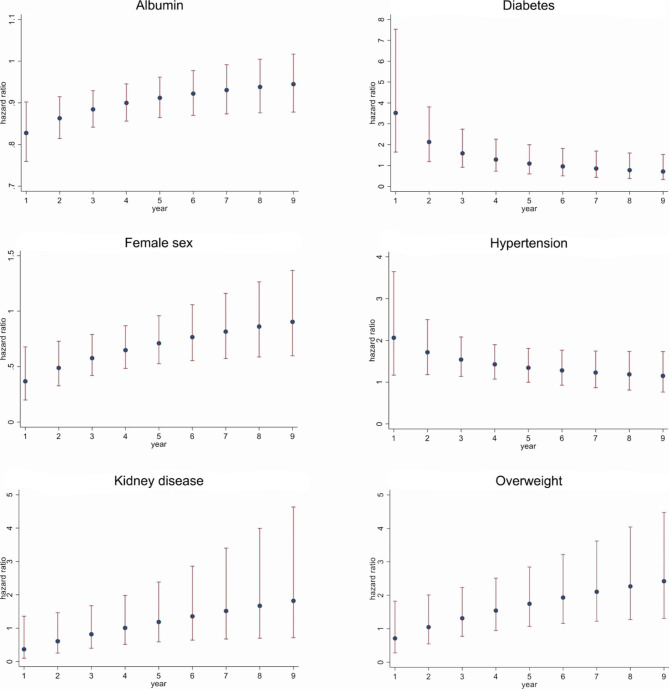
Variation of HRs over time for variables violating the proportional hazard assumption (bars indicate 95%-confidence intervals)

A Cox proportional hazard regression model was derived for the general population (Table 2). The lowest Akaike information criterion was obtained with a model including interaction terms of sex, overweight, age (in overweight subjects), albumin, hypertension, diabetes and renal disease with time. As time variables, we alternatively considered t, the natural logarithm of t and the square root of t. The AIC-values were 2625.63, 2618.664 and 2621.889, respectively. Therefore, the model including interactions with the natural logarithm of t was retained. The variations of HRs over time for these variables are depicted in Fig. 1. Moreover, two separate gender-stratified models were calculated (Tables 3 and 4). The Chi-squared test revealed significant differences in the HRs between males and females for smoking (χ^2^ = 5.22, p = 0.022, with a stronger effect in women) and osteoporosis (χ^2^ = 5.71, p = 0.017, with a stronger effect in men).

**Table 2:** Cox proportional hazard regression model (AIC = 2618.664)

	HR	p-value	95% C.I.			
Female gender* (↑)	0.65	0.004	0.48	-	0.87	
Overweight* (↑)	1.54	0.082	0.95	-	2.51	
Age in overweight subjects* (↑)	1.13	< 0.001	1.10	-	1.16	
Age in normal weight subjects	1.17	< 0.001	1.14	-	1.21	
Albumin* (↑)	0.90	< 0.001	0.86	-	0.95	
Smoking	2.24	0.001	1.37	-	3.65	
Hypertension* (↓)	1.43	0.015	1.07	-	1.89	
Diabetes* (↓)	1.29	0.383	0.73	-	2.26	
Osteoporosis	2.03	0.034	1.06	-	3.92	
Renal disease* (↑)	1.01	0.982	0.51	-	1.98	
History of cancer	3.83	< 0.001	2.34	-	6.25	

* indicates variables for which the proportional hazard assumption was violated based on the Akaike information criterion. The respective estimates refer to a follow-up time of 4 years

↑ or ↓: HR increases (↑) or decreases (↓) over time

**Table 3:** Cox proportional hazard regression model in males (AIC = 1210.9)

	HR	p-value	95% C.I.		
Overweight (↑)	2.05	0.039	1.04	-	4.06
Age in overweight subjects	1.12	< 0.001	1.08	-	1.16
Age in normal weight subjects	1.17	< 0.001	1.12	-	1.22
Albumin	0.92	0.022	0.86	-	0.99
Smoking	1.59	0.133	0.87	-	2.95
Hypertension (↓)	1.29	0.229	0.85	-	1.95
Diabetes	2.10	0.013	1.17	-	3.79
Osteoporosis	11.42	0.001	2.59	-	50.31
Renal disease (↑)	0.78	0.747	0.18	-	3.40
History of cancer	7.24	< 0.001	3.80	-	13.79

↑ or ↓: variables violating the proportional hazard assumption based on the Akaike information criterion, with the HR increasing (↑) or decreasing (↓) over time. The hazard ratios reported for these variables refer to a follow-up time of 4 years

**Table 4:** Cox proportional hazard regression model in females (AIC = 1107.9)

	HR	p-value	95% C. I.		
Overweight	1.10	0.798	0.52	-	2.32
Age in overweight subjects (↑)	1.16	< 0.001	1.11	-	1.21
Age in normal weight subjects (↓)	1.19	< 0.001	1.15	-	1.24
Albumin	0.89	0.004	0.84	-	0.97
Smoking (↓)	5.29	< 0.001	2.32	-	12.08
Hypertension	1.43	0.085	0.95	-	2.15
Diabetes (↓)	0.05	0.199	0.0006	-	4.66
Osteoporosis	1.48	0.324	0.68	-	3.23
Renal disease	1.35	0.574	0.47	-	3.86
History of cancer	3.44	0.002	1.57	-	7.53

↑ or ↓: variables violating the proportional hazard assumption based on the Akaike information criterion, with the HR increasing (↑) or decreasing (↓) over time. The hazard ratios reported for these variables refer to a follow-up time of 4 years

#### Fitting of the old model and Kaplan-Meier curves

As comparison, we fitted the model obtained in our previous analysis [7] to the complete follow-up data (Table 5). Female gender (HR 0.64, 95% C.I. 0.48–0.85, p = 0.002), hypertension (HR 1.48, 95% C.I. 1.12–1.95, p = 0.006) and osteoporosis (HR 2.33, 95% C.I. 1.24–4.37, p = 0.008) still showed statistically significant independent associations with all-cause mortality. However, there was no longer significant non-linearity in the estimated effect of age.

**Table 5:** Fitting of the model from Gutzwiller et al. [7]

	HR	p-value	95% C.I.			
Female	0.64	0.002	0.48	-	0.85	
Age-74	1.153	< 0.001	1.124	-	1.183	
(Age-74)*(Age-74)	1.00	0.502	0.99	-	1.01	
Hypertension	1.48	0.006	1.12	-	1.95	
Diabetes	1.46	0.157	0.86	-	2.45	
Osteoporosis	2.33	0.008	1.24	-	4.37	

Age was centered at 74 years to remove the high initial collinearity between the linear and the quadratic term of age.

**Fig. 2:**
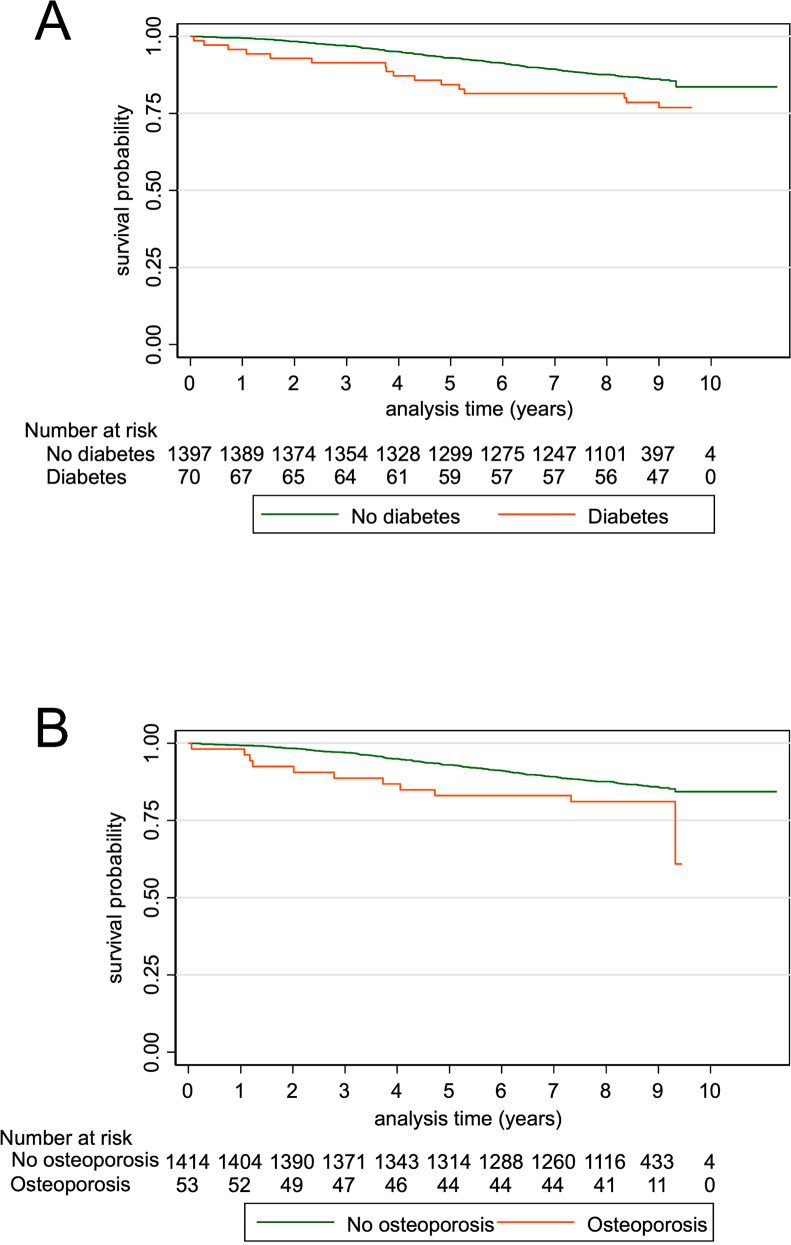
Kaplan-Meier curves for survival in patients with and without diabetes (**A**) and osteoporosis (**B**), respectively

The Kaplan-Meier curves show survival over time among participants with and without T2DM (Fig. 2A) and osteoporosis (Fig. 2B), respectively.

## Discussion

This paper presents mortality-associated risk factors in a cohort of 1467 subjectively healthy, community-dwelling Swiss adults aged 65 or more, over a median follow-up period of 8.79 years. On December 31^st^ 2019, we recorded 208 deaths, of which 106 were males and 102 females.

Among the variables included in the final model, significant predictors of all-cause mortality included female gender, age, albumin level, active smoking, arterial hypertension, osteoporosis, and history of cancer. Slight deviations from this general model were observed when the analysis was stratified by gender. Nevertheless, our results seem clinically meaningful and appear to be in line with similar findings in the literature.

The results of this updated analysis also confirmed the role of T2DM as long-term risk factor for mortality. In 2015, it was estimated that almost 10% of the global adult population was affected by diabetes mellitus, 90% being T2DM [15]. Swiss prevalence of T2DM is slightly lower (6.3%), with a slightly higher rate in males [16]. The Global Burden of Disease Study 2013 identified diabetes mellitus as the ninth major cause of reduced life expectancy [10]. Micro- and macrovascular complications occur in about 50% and 27%, respectively, of T2DM patients [15]. In particular, microvascular complications in patients with diabetes are not only the cause of retinopathy, nephropathy or neuropathy, but they also play a major role in the onset of cardiovascular complications [11]. It is well established that gonadal hormones deficiency affects T2DM pathophysiology in a sex-specific manner [17]. However, while oestrogen deficiency in menopausal women is usually prevented by the wide use of oestrogen replacement therapy, the male testosterone deficiency is less often replaced, thus increasing the progression rate from prediabetes to T2DM in males. For the decreasing hazard ratio of diabetes during follow-up, we hypothesize the following two reasons. First, mortality among diabetics may have been driven by those with severe disease in the first phase of follow-up, and, second, a certain proportion of participants who were disease-free at baseline might have developed diseases during follow-up. Both processes would have steadily reduced the initial risk difference between diabetics and non-diabetics.

Active smoking and history of cancer resulted significant predictors of mortality in elderly Swiss adults. The lack of statistical significance of these two variables in our previous analysis [7] was likely due to the relatively short initial surveillance period; it was concluded that, over a 3-year follow-up, comorbidities other than neoplasia played a more important role in mortality. The same line of reasoning was applied to smoking. However, our updated results conform with those obtained in similar cohort studies demonstrating that smoking [6, 18, 19] and previous history of cancer [19] were risk factors associated with mortality over a follow-up timeframe ranging between 3-to-8 years. Although absolute mortality rates might be similar in both genders due to inequalities to baseline mortality rates, previous cohort studies postulated that women may be more sensitive than men to some of the deleterious effects of smoking [20]. A recent metanalysis performed on more than 2 million subjects estimated 1.25 higher risk for coronary heart disease in smoking females with respect to men [21]. Revealing whether the mechanisms underlying the sex difference in smoking-related mortality are biological or related to differences in smoking behaviour between men and women goes beyond the scope of this analysis. However, we provided adding evidence to the existing literature that should prompt healthcare providers to embrace even stronger actions to tackle this modifiable risk factor, especially in the female population.

Not surprisingly, increasing age was confirmed as risk factor associated with mortality. Age is a well-known risk factor leading to accumulation of multiple comorbid conditions [19, 22]. This will inevitably reflect into an increased likelihood of dying over long follow-up periods. Previous studies also demonstrated association between age, sex and several different predictors of mortality [6].

In order to compare homogeneous results, we fitted the model obtained in 2018 [7] to the complete follow-up data. Osteoporosis and hypertension were consistently associated with increased all-cause mortality. On the other hand, female gender was found to be a protective factor. Interestingly, diabetes and the square term of age were no longer statistically significant when considering data from the entire follow-up. The decrease in the estimated effect of diabetes can be explained by the observed decrease in the importance of this baseline predictor with increasing time of follow-up.

In 2019 more than half million Swiss people lived with a diagnosis of osteoporosis, of whom almost 80% were women [23]. The prevalence of osteoporosis in the Swiss population amounts to 6.1%, which is similar to the average prevalence in other European countries (5.6%) [23]. Osteoporosis is a common cause of bone fractures, especially of the hip joint or of the vertebrae. In the event of an osteoporotic fracture, these may lead to serious disability, reduced quality of life and motility, chronic pain, and increased premature mortality [8, 9]. The mortality associated with a fracture among Swiss adults aged 50 years or older was estimated to be 107 per 100,000 person years, just slightly below the European average (116:100,000) [23]. It was also postulated that the number of fracture-related fatalities was comparable to some of the most common causes of death such as lung cancer, diabetes or chronic respiratory diseases [23]. In our population, osteoporosis caused a two-fold increased risk of death, even after adjustment for other factors. Describing the causal relationship between osteoporosis and mortality goes beyond the purposes of this study. However, we believe osteoporosis to be a reliable proxy of pathological fractures, which may increase mortality through several either direct or indirect pathways [24].

Hypertension is a universally acknowledged risk factor for mortality, especially in the elderly population. Worldwide, elevated arterial blood pressure is responsible for 7.6 million deaths annually [12]. On average, one in two cardiovascular or cerebrovascular events are attributable to hypertension [12]. Although elevated arterial blood pressure can be controlled with standard pharmacological therapy in most of the subjects, there are still 5% of patients being refractory to combination of therapies [25].

As a recent review reported [26], the 2011 European Commission report highlighted a two-times higher mortality rate in men than women. It is speculated that poor lifestyles and untreated, preventable risk factors are accounting for half of premature deaths in men. Moreover, men typically neglect pain and disease, and they are less prone to engage in routine check-ups with respect to women [26]. Besides the sociological and epidemiological aspects, the pure biological sex is – per se – a very important modifier of human physiology, disease predisposition, manifestation, and response to treatment via genetic, epigenetic, and hormonal regulations [26]. Taken together, these considerations apply to a broad range of pathological conditions [26] and, no less, to the Swiss population.

Although the SENIORLAB study [13] collected more than 110 laboratory parameters, we decided to include in this analysis only those for which there was already an established evidence in the literature regarding their association with mortality. In particular, serum albumin is a proxy of nutritional status, which is a well-established predictor of mortality in community-dwelling older adults [27, 28]. One reason for that might be the association between hypoalbuminemia (a proxy of catabolic state) and worse recovery following acute pathologies [28]. In our analysis, we confirmed the protective effect of adequate albumin levels, which are inversely related to the mortality risk (HR, 0.90; 95% CI, 0.86–0.95, p < 0.001).

The present study is characterized by several strengths which support our findings. First, this study is based on a prospective design, with demographic, health-related and laboratory data of a relatively wide population-based cohort of community-dwelling older adults, providing adequate statistical validity to the results. Second, the updated follow-up of 8.79 years represents, to our knowledge, the longest follow-up time frame for elderly Swiss people. Third, we reported a broad range of potential risk factors, validated across multiple studies. Fourth, none of the patients was lost to follow-up and the vital status could be assessed for the entire cohort at the end of the study period. Lastly, follow-up was concluded on the 31st December 2019, before the first case of SARS-CoV-2 infection could be registered in Switzerland. Therefore, the reported results are not affected by the effects of Coronavirus pandemic that hit Europe since the beginning of 2020.

However, we also acknowledge some limitations to the generalization of our results. The most important weakness of this study is represented by recall bias of subjects included in the sample, thus resulting in potential underestimation of comorbidities. Another limitation is the absence of indicators of physical activity. In fact, it is considered an important factor linked to overall morbidity and mortality in older adults and could represent an informative predictive parameter of better outcomes in elderly subjects [29]. A further limitation is the lack of information regarding the socioeconomic status of the participants to the study. As reported in the original paper by Risch et al. [13] describing the study design, study participants were contacted through newspaper advertisements, various clubs, and associations with high proportions of healthy elderly members. Therefore, it is possible that this recruitment led to an over-representation of participants with medium or high socioeconomic status. Lastly, we acknowledge the lack of individual follow-up of risk factors and health status. All information was only collected at baseline only; however, this limitation only applies to a restricted set of variables (e.g., smoking, albumin…). On the other hand, most chronic conditions present at baseline remained and the occurrence of new diseases during the follow-up period does not invalidate the prediction made according to baseline information.

Identification of predictors of mortality in community-dwelling older adults goes beyond simply enhancing medical knowledge: it is deeply associated with practical implications. Appropriately targeting modifiable risk factors and preventing comorbidity onset will almost certainly promote a better quality of life for the elderly. In the light of the results obtained in our study, controlling diabetes, hypertension and the nutritional status with adequate dietary regimens and calcium supplementation for prevention of onset of osteoporosis represent two potential cost-effective interventions with a dual effect: ensuring healthy ageing, while reducing the economic burden of the elderly.

## Conclusions

By extending the follow-up on a sample of subjectively healthy, community-dwelling, older Swiss adults we confirmed the role of osteoporosis, T2DM and hypertension as major risk factors for all-cause mortality. Low albumin level, smoking and history of cancer have emerged as new predictors of mortality over an extended follow-up period.

## Data Availability

The datasets generated and/or analysed during the current study are not publicly available due to privacy issue but are available in anonymized form from the corresponding author on reasonable request.

## List of abbreviations

CI: confidence interval
CRP: C reactive protein
HOMA-IR: insulin resistance index
HR: hazard ratio
IQR: interquartile range
SD: standard deviation
T2DM: type 2 diabetes mellitus
